# Investigating the Effects of Mechanical Stimulation on Retinal Ganglion Cell Spontaneous Spiking Activity

**DOI:** 10.3389/fnins.2019.01023

**Published:** 2019-09-27

**Authors:** Marica Marrese, Davide Lonardoni, Fabio Boi, Hedde van Hoorn, Alessandro Maccione, Stefano Zordan, Davide Iannuzzi, Luca Berdondini

**Affiliations:** ^1^LaserLab, Department of Physics and Astronomy, Vrije Universiteit Amsterdam, Amsterdam, Netherlands; ^2^NetS^3^ Laboratory, Neuroscience and Brain Technologies Department, Fondazione Istituto Italiano di Tecnologia, Genova, Italy

**Keywords:** mechanical stimulation, high-density electrophysiology, retina, neural circuits, viscoelasticity, spontaneous activity

## Abstract

Mechanical forces are increasingly recognized as major regulators of several physiological processes at both the molecular and cellular level; therefore, a deep understanding of the sensing of these forces and their conversion into electrical signals are essential for studying the mechanosensitive properties of soft biological tissues. To contribute to this field, we present a dual-purpose device able to mechanically stimulate retinal tissue and to record the spiking activity of retinal ganglion cells (RGCs). This new instrument relies on combining ferrule-top micro-indentation, which provides local measurements of viscoelasticity, with high-density multi-electrode array (HD-MEAs) to simultaneously record the spontaneous activity of the retina. In this paper, we introduce this instrument, describe its technical characteristics, and present a proof-of-concept experiment that shows how RGC spiking activity of explanted mice retinas respond to mechanical micro-stimulations of their photoreceptor layer. The data suggest that, under specific conditions of indentation, the retina perceive the mechanical stimulation as modulation of the visual input, besides the longer time-scale of activation, and the increase in spiking activity is not only localized under the indentation probe, but it propagates across the retinal tissue.

## Introduction

Eukaryotic cells are constantly subjected to biomechanical interactions with the extracellular environment. Mechanical forces play a fundamental role in many different aspects of cell birth, life, and death. Starting from stem cells, external forces and environmental mechanical constraints are vital in order to trigger the differentiation process and to define the cell fate both in embryonic development (Vogel and Sheetz, 2006; Reilly and Engler, 2010; Yim and Sheetz, 2012) and in adulthood (Vining and Mooney, 2017) with the unprecedented possibilities to design biomechanical therapies for *in vivo* tissue regeneration. Not only single cells, but also entire organs, such as heart (Terracio et al., 1988; Franz et al., 1989; Kohl et al., 2006) and lungs (Wirtz and Dobbs, 2000), are strongly affected by mechanical forces that can change their cellular organization, structure, functionalities, and electrophysiological signaling.

Furthermore, also biomechanical forces exerted on cells in the brain have important, yet partially understood effects on the physiological development and functional behavior of brain circuits (Barnes et al., 2017). Neurons and glial cells are embodied in brain circuits, and intrinsically experience physical cues due to their surrounding physical world, which include, among others, electrochemical gradients and mechanical stresses (Tyler, 2012; Franze, 2018). At the cellular level, mechanotransduction can influence several cellular processes in the brain, including differentiation, survival, proliferation, and migration, thus contributing to its physiological or pathophysiological development (Ingber, 2003). The role of cellular mechanotransduction has also been associated with cellular and sub-cellular injuries that may ultimately lead to the diffusion of pathological damage in traumatic brain injury (Hemphill et al., 2015). Additionally, biomechanical forces are involved in cerebral cortical folding as well as in folding abnormalities in neurodevelopmental brain disorders.

Unraveling the functional role of biomechanical forces in brain circuits is, therefore, a crucial research topic in neuroscience. However, the investigation of contact forces (e.g., shear, compression, or tension) in modulating brain circuit’s physiological functions requires technology advances. In this direction, here, we propose an experimental platform to probe the electrophysiological effects of controlled contact forces in brain circuits with an unprecedented resolution. This technique offers a unique opportunity to investigate the effects of mechanical stimulation on the electrophysiological activity of neuronal tissue, such as cell culture networks, brain slices, and retina samples, at the single-cell resolution and over a wide portion of biological tissue. To demonstrate the probing performances of this technique, here we investigated contact force effects on the electrophysiological responses to visual stimuli in explanted mice retinas.

The retina is the light-sensitive tissue devoted to convert and pre-process variations of light intensity into spike trains that are decoded by downstream areas of the brain to generate visual perception. While the light-sensitive properties of the retina are widely studied because they represent the principal and fundamental characteristic of the tissue, its mechanical and mechanosensitive properties are currently under-investigated. The retina, however, is constantly subjected to mechanical stresses induced by intraocular pressure, suction forces pulling the outer retinal surface (Marmor, 1993), and inertial forces applied by the vitreous body upon physical shocks, traumatic events, or rapid eye/head movements (Wygnanski-Jaffe et al., 2007). Mechanical forces, as tension, hydrostatic pressure, shear, stretch, compression, and torsion provide cells with essential cues about the surrounding physical world and ultimately impact on cell viability. Specifically, in the context of the retina, pathologies such as axial myopia and glaucoma may arise from prolonged mechanical stress (Bonomi et al., 2001; Tan et al., 2006).

Interestingly, to cope with and transduce a mechanical signal, several populations of mouse retinal cells, as Müller cells, Retinal Ganglion Cells (RGCs) and photoreceptors are equipped with mechanoreceptors (Sappington et al., 2015) – a mechanically gated family of ion channels activated by signal pressure or tractional forces. Defective or impaired activity of these channels has functional implications on retina physiology by affecting reception and integration of synaptic signals (Manivannan and Suresh, 2012), involving Müller cells K^+^ clearance (Skatchkov et al., 2006). The presence of such receptors suggests that the retina might collect sensory information not limited to the visual scene but also reflecting the current state of the tissue (Križaj, 2016). Indeed, elevated intraocular pressure regulates RGCs excitability (Ryskamp et al., 2011). Furthermore, mechanical stimulation of Müller cells − the major astrocytic population in the retina − induces waves of calcium (Agte et al., 2017) that modulate the RGCs spiking response to white/black flash cycles in mouse retinas (Munsaka et al., 2009) and are known to be sensitive to mechanical stretches (Lindqvist et al., 2010). In addition, in a pivotal study, Grüsser et al. (1989) reported the induction of phosphenes (i.e., the perception of variations in light intensity when no visual stimulation occurs) upon application of physical pressure through stretching cat eyeballs. Specifically, after a latency of 0.2–4.0 s, the mechanical stimulation activated ON RGCs and inhibited OFF RGCs during the stimulation time-window and determined transient responses in a subpopulation of ON RGCs upon pressure release.

Similarly, the exploitation of retinal mechanosensitive properties has been recently proposed as a new technological approach for prosthetic purposes (Rountree et al., 2018). Indeed, it was shown that mechanical stimuli induced by pulses of a physiological solution injected within the internal layer of rat retina could elicit localized RGCs responses, with amplitude and latencies comparable to those observed with direct light stimulation, even in retinas with degenerated photoreceptors. Finally, in micro electroretinograms (microERGs) experiments, the application of extra weight onto the retina to improve the coupling between RGCs and electrodes affected the response to full-field flashes, although not significantly (Fujii et al., 2016).

To disentangle the mechanosensitive features that might influence visual information encoding in the retinal circuit, in our experiments we mechanically stimulated the retina with micrometer precision from the side of the photoreceptor layer and simultaneously recorded the spiking activity of thousands of RGCs at the pan-retinal scale. To do so, we combined a depth-controlled force transducer with a high-density multi-electrode array (HD-MEAs) in order to record extracellular RGCs spiking activity while photoreceptors were mechanically stimulated. The electrophysiological acquisition system provides sub-millisecond recordings from a 64 × 64 grid of 42 μm-spaced electrodes covering an area of 2.67 mm × 2.67 mm, which is comparable to the extension of a mouse retina.

Simultaneous use of these two technologies allows one to perform systematic studies on two interconnected topics: on the one hand, the mechanical properties of the retina can be characterized through tissue microindentation; on the other hand, our approach can reveal how a localized mechanical stimulation on the photoreceptor layer may trigger or affect the spontaneous spiking activity of RGCs, i.e., the output neurons of the retina. We present here, for the first time, a depth-controlled mechanical characterization of the retinal tissue that confirms and extends the work of Franze et al. (2011) by highlighting the viscoelastic nature of the sample. We further demonstrate that, under specific indentation conditions, mechanical stimulation can induce a response in a subset of RGCs, suggesting that the retina integrates the effects of biomechanical forces when encoding visual inputs.

## Materials and Methods

### Ethics Statement

All the experiments were performed in accordance with the guidelines established by the European Community Council (Directive 2010/63/EU of 22 September 2010). All procedures involving experimental animals were approved by the institutional IIT Ethic Committee and by the Italian Ministry of Health and Animal Care (Authorization number 110/2014-PR, December 19, 2014).

### Experimental Setup

**FIGURE 1:**
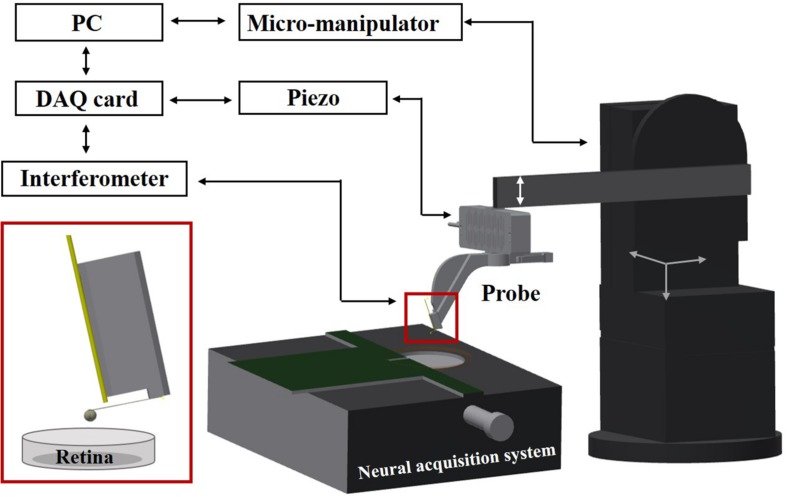
Schematic view of the setup. A ferrule-top probe is equipped with an optical fiber for interferometric readout of the cantilever and with a spherical tip to indent the sample. The probe is mounted on the Z-piezoelectric actuator, which is solidly attached to an XYZ manipulator. Retina samples are submerged in the perfusion chamber and fixed on HD-MEAs based on a high-impedance CMOS chip.

The setup consists of an indentation arm, a chip-based electrophysiological platform, and a sample holder (see Figure 1). The indentation arm includes an XYZ micromanipulator (PatchStar, Scientifica, United Kingdom), a Z-piezoelectric actuator (PI p-603.5S2, Physik Instrumente) and a ferrule-top indentation probe connected to an interferometer (OP1550, Optics11, The Netherlands). The indenter is based on a micro-machined cantilever spring, operating as a force transducer. An extensive description of the probe fabrication and the indentation setup can be found elsewhere (Gruca et al., 2010; Van Hoorn et al., 2016). A custom- written LabVIEW software (National Instruments) is used to process signals and to control the devices through a data acquisition card (PCIe-6361, National Instruments). Retina samples are submerged in the perfusion chamber and fixed on a HD-MEAs. The system is placed on a vibration isolation table to minimize external noise. All the experiments are performed at room temperature, using indentation depth-control mode (Antonovaite et al., 2018).

### Indentation Protocol for Mechanical Characterization

To probe viscoelasticity of retina samples, depth-controlled frequency sweep measurements have been performed at small oscillation amplitude and different frequencies. Typically, dynamic indentations (maps) consisted of a loading part up to 15 or 20 μm with 2 μm/s indentation speed, followed by 10 s stress relaxation period and a series of small sinusoidal oscillations of 0.3 μm at three different frequencies: 0.1, 1, and 10 Hz. For indentation maps, we selected cantilevers with ∼0.45 N/m spring constant, calibrated according to Beekmans and Iannuzzi (2015), and bead radius of 57 and 73 μm, measured via optical microscope.

Indentations were performed in an arbitrary region of the retina in parallel lines, with a distance between two adjacent locations of 50 μm, which assured that the deformed areas do not overlap. The apparent storage and loss moduli were calculated as:

(1) $$K^{\prime }=\frac{E^{\prime }}{1-v^{2}}=\frac{F_{0}}{h_{0}}\,c\,o\,s\,\left(\Phi \right)\,\frac{\sqrt{\pi }}{2}\,\frac{1}{\sqrt{A}}$$

(2) $$K^{{}^{\prime \prime }}=\frac{E\,"}{1-v^{2}}=\frac{F_{0}}{h_{0}}\,\left(\Phi \right)\,\frac{\sqrt{\pi }}{2}\,\frac{1}{\sqrt{A}}$$

where *E*′ and *E*″ are the storage and the loss modulus, respectively, *F*_0_ is the amplitude of the oscillatory load, *h*_0_ is the amplitude of the oscillatory-indentation, ν is the Poisson’s ratio of compressibility (assumed to be equal to 0.5, which corresponds to an incompressible material), Φis the phase lag between the recorded indentation and load oscillations, and *A* = π*a*^2^ is the contact area between the sphere and the sample. The contact radius is estimated as $a=\sqrt{R\,h}$ where *R* is the radius of the sphere and *h* is the indentation depth. For the final data analysis, three maps of two retinae were used. Normality of data distribution was tested with the Shapiro–Wilk test. Since the majority of the dataset were non-normally distributed, the non-parametric Kruskal–Wallis ANOVA test was used to compare data samples. All statistical analyses were performed with Statistics and Machine Learning Toolbox (version 2018a, The Mathworks, Natick, MA, United States).

### Retina Electrophysiology

Twelve hours dark-adapted male mice (6 weeks old C57BL/6) were barely anesthetized with CO2 and subsequently killed by cervical dislocation. As described in Hilgen et al. (2017a), Maccione et al. (2014), after eyeballs enucleation, the retina was extracted by accurately removing all the surrounding tissues such as the cornea, crystalline, sclera, and vitreous. Once isolated, the retina was faced down onto a pre-conditioned HD-MEAs (its reservoir was filled with Neurobasal for 2 h at 37°) putting the retinal ganglion layer in contact with the surface of the electrodes and leaving the photoreceptor layer exposed to the indentation probe. A perfusion line, supplied by a peristaltic pump (∼1 ml/min), ensured a constant flow of a media composed by AMES’s medium (Sigma - Merck KGaA, Darmstadt, Germany) with 1.9 g/L of sodium bicarbonate equilibrated with carboxigen (95% O2 and 5% CO2). Recordings of RGCs extracellular activities were acquired through the BioCam4096 platform with 4096 Arena chips (3Brain AG, Wädenswil, Switzerland), which consist of a 64 × 64 grid of square microelectrodes (21 μm × 21 μm, pitch 42 μm) covering an area of 2.67 mm × 2.67 mm (HD-MEAs). The raw extracellular traces, sampled at 7.1 kHz/electrodes, were digitized at 12-bit resolution and stored for off-line analysis upon application of a low-pass filter (3.5 kHz) using Brainwave software. Spikes in extracellular traces were detected and sorted exploiting the redundant information of spatially adjacent electrodes (Hilgen et al., 2017b). Only single units exhibiting at least 0.1 spike/sec were considered for subsequent analysis. Upon application of this filtering procedure, the resulting dataset consists of multiple hundreds of single units per single retina.

### Experimental Protocol for Electrophysiological Characterization

**FIGURE 2:**
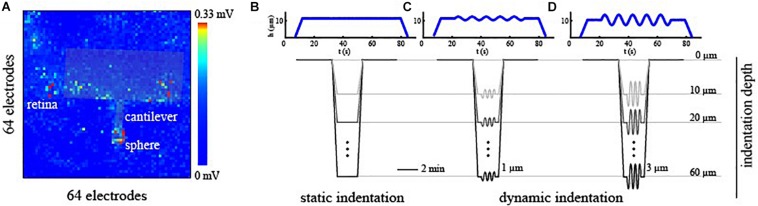
**(A)** Alignment of the indentation probe over the electrode grid of the HD-MEAs. The contour of the indentation probe was detected by looking at non-saturating channels upon far-red light stimulation. **(B–D)** Mechanical stimulation profile as a function of time for static indentation [no frequency sweep, **(B)**], small oscillation amplitude [h_0_ = 1 μm, **(C)**], and mild oscillation amplitude [h_0_ = 3 μm, **(D)**] types of stimuli. Exemplary mechanical profiles are plotted in blue for an indentation depth of 10 μm. Mechanical indentations were then performed at progressively deeper levels ranging from 10 to 60 μm from the contact point.

To correlate the effects induced by the mechanical stimulation of the photoreceptor layer with the spiking activity of RGCs acquired with HD-MEAs, two pieces of information were essential: the time-window of mechanical stimulation and an estimation of the indentation position respect to the electrode grid. To identify the stimulation time-window, we used a Raspberry Pi device (Raspberry Pi Foundation) that, through custom python algorithms, memorizes the timing of the triggers generated by the indenter at the beginning and the end of the stimulation phase relative to the starting point of the HD-MEAs recording. Next, this information was incorporated into the spike-trains allowing an *a posteriori* alignment of the indentation time-interval within the recorded spiking activity. To navigate the indentation probe onto the electrode matrix and estimate the indentation position, we shined a far-red light onto the retina for ∼5 s. This procedure triggers light-induced signal saturation in all the electrodes of the HD-MEAs except for those located in the region shadowed by the indentation probe (see Figure 2A). Upon selecting through visual inspection, the time-frame at which the difference between non-saturating and saturating electrodes was maximally noticeable, we manually detected the contour of the indentation probe. Next, we estimated the indentation position as the electrode corresponding to the center of the indentation sphere located on the indentation probe.

To obtain a direct readout of electrical signaling upon mechanical deformation, we used large spheres (∼250 μm beads), and we probed specific local regions of the retina at different amplitudes and indentation depths. Each sample was probed through three controlled indentation protocols, described in Figure 2, that differed in the dynamic of the indentation over time. Specifically, once the probe reached the desired depth, we tested the retinal mechanical and electrophysiological response to a steady and continuous displacement (Figure 2B) and to small (Figure 2C, h_0_ = 1 μm) or mild (Figure 2D, h_0_ = 3 μm) oscillations amplitude at 0.1 Hz. As summarized in Figures 2B–D, these protocols were performed at depths ranging from 10 to 60 μm for each experimental session and were interleaved with a minimum of 2 min of basal recording to avoid the application of prolonged stresses to the retinal tissue. Prior to indentation protocols, the spontaneous activity of the RGCs was recorded for 5 min to obtain a baseline reference of retinal activity and, after that, the response of RGCs has been visually stimulated with a sequence of black and white flashes (see ON-OFF classification section) to probe their preferential response. Next, the indentation probe approaches the tissue, and its position was estimated.

### Data Analysis

Before each indentation phase, we recorded 120 s of basal spiking activity, which we used to determine the reference firing regime of RGCs before mechanical stimulation. For both basal and indentation phases, the mean firing rate of each single RGCs was computed by quantifying the number of spikes occurring in a 2 s time-interval sliding over the recording period with steps of 10 ms. Next, the mean firing rate was normalized through z-scoring to ensure a comparison over standardized firing rates. The correlation matrix between the normalized firing rate was then computed for both the basal and indentation phases.

### Clustering of Similar Spiking Activity

To highlight the modulatory effects exerted by mechanical indentation onto RGCs spiking activities, we clustered the firing rate of single RGCs according to their variations over time. A standard dendrogram clustering procedure was applied to the normalized firing rate (computed every 10 ms over a 2 s sliding window) of the indentation phase using the absolute value of Pearson correlation as similarity metric (Hierarchical clustering [(scipy.cluster.hierarchy) — SciPy v1.1.0 Reference Guide]. We then selected an optimal number of clusters within the range 4 to 60 through maximization of the silhouette score (sklearn.metrics.silhouette_score — scikit-learn 0.20.0 documentation). Next, we reordered the correlation matrix of the basal phase according to the clustering obtained during the indentation phase to prove that the clustered RGCs were not correlated during the basal time-window and thus that correlations in RGCs spiking activity arise from mechanical stimulation.

### Classification of ON-OFF RGC Cell Types

To assess whether subpopulations of RGCs are most sensitive to the effects of mechanical stimulation, we first had to identify functional RGC cell types based on their preferential responses to visual stimuli. To do so, at the beginning of the experiment, we presented to the retina a sequence of alternating white/black full-field flashes (0.0–0.2 cd/m2) that allow characterizing the RGCs spiking response properties. To distinguish between ON, OFF and ON-OFF RGCs we computed the Bias Index (BI) as (A_*w*_−A_*b*_)/(A_*w*_ + A_*b*_), where A_*w*_ and A_*b*_ are the amplitudes of the peak response, with respect to the basal level of activity, to the white and black flashes, respectively, (Carcieri, 2003). RGCs were marked ON if BI>0.3, OFF if BI<−0.3, and ON-OFF otherwise. Cells whose peak firing rate was one standard deviation below the mean basal firing rate were assigned to the non-classified cluster (NC).

### Decision Tree Classifier

To disentangle crucial physical and electrophysiological predictors that can determine the modulation of spontaneous spiking activity upon mechanical stimulation, we applied a Decision Tree Classifier (scikit-learn: machine learning in Python — scikit-learn 0.20.2) on all trials. This machine learning model learns how to classify binary outcomes as successful and unsuccessful indentations by building a tree of if-then-else relations among the predictors provided. In our scenario, the predictors are: the contact area of the indentation probe, the strain, the pressure, the depth of the indentation, the local density of RGCs below the indentation point (d5, number of cells within five electrode distance) and the local firing rate of RGCs point below the indentation point in basal condition (r5, average over cells within five electrode distance). Once the decision tree classifier is trained, the importance of each feature is computed as the normalized Gini importance. To ensure unbiased results, we obtained averaged values by repeating the procedure 1000 times with different initial conditions.

## Results

The primary purpose of this study was to design a new experimental setup able to combine a HD-MEAs with ferrule top indentation to provide insights about electromechanical coupling in retina tissue.

### Characterization of Mechanical Properties of the Retina Tissue

**FIGURE 3:**
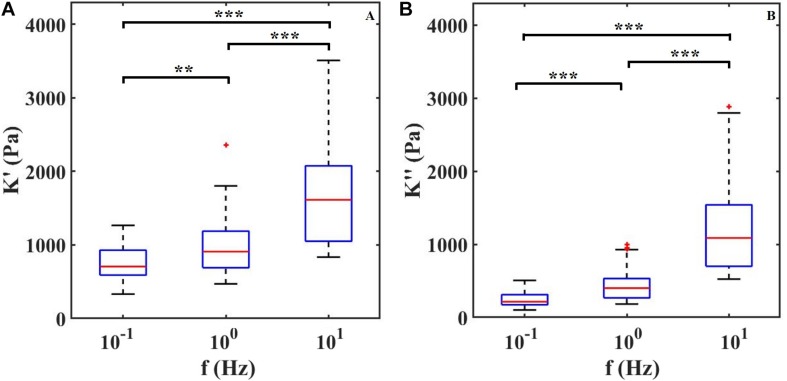
**(A)** Apparent storage (*K*′) and **(B)** loss (*K*″) moduli plotted as a function of indentation frequency. Each column contains data of two retinae and around 30 measurements per frequency. ^∗∗^*P* < 0.01 and ^∗∗∗^*P* < 0.0005 by Kruskal–Wallis test.

To confirm that ferrule-top depth-controlled dynamic indentation is capable of capturing the viscoelastic nature of the retina even when combined with HD-MEAs, we performed three indentation maps on two different samples. In Figure 3, we report the dynamic response of the retina, in terms of apparent storage and loss moduli over the frequency range of 0.1–10 Hz. The frequency sweep data show a stiffening of the tissue with increasing indentation frequency for both the apparent loss and storage moduli. For instance, the averaged storage modulus, pooled from all the indented locations, increases from 0.7 kPa at 0.1 Hz to 1.7 kPa at 10 Hz.

With the dynamic indentation, one can define the ratio between the loss and storage modulus, known as loss tangent, *Tan*(Φ), which provides the relation between viscous and elastic components.

(3) $$T\,a\,n\,\left(\Phi \right)=\frac{E^{\prime \prime }}{E^{\prime }}$$

**FIGURE 4:**
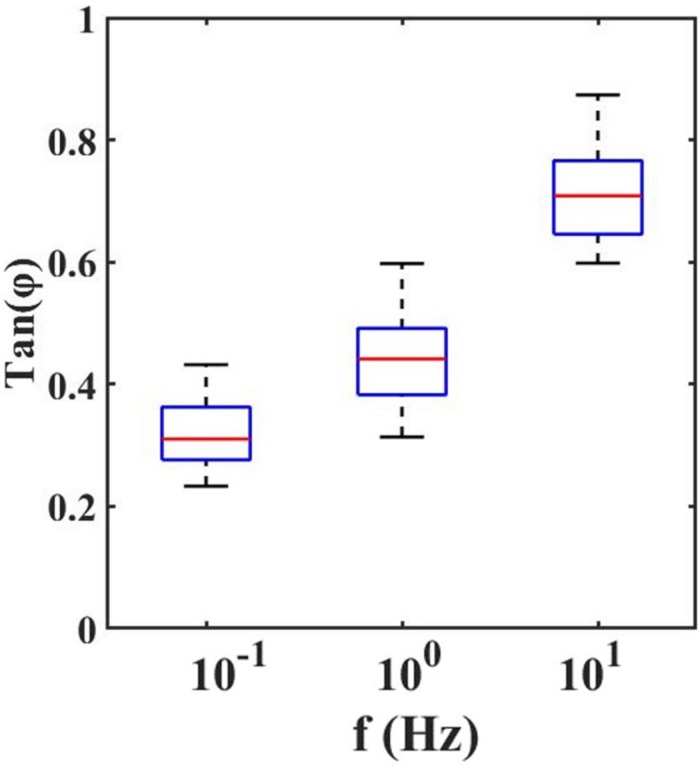
Loss Tangent: the tangent of the phase angle between load and indentation as a function of frequencies.

From the loss tangent, in Figure 4, one can observe that when the tissue is stimulated at 10 Hz, the viscous contribution is higher if compared to low frequencies and thus the tissue has higher damping capability.

### Mechanical Indentation Affects the Spontaneous Activity of Retinal Ganglion Cells

**FIGURE 5:**
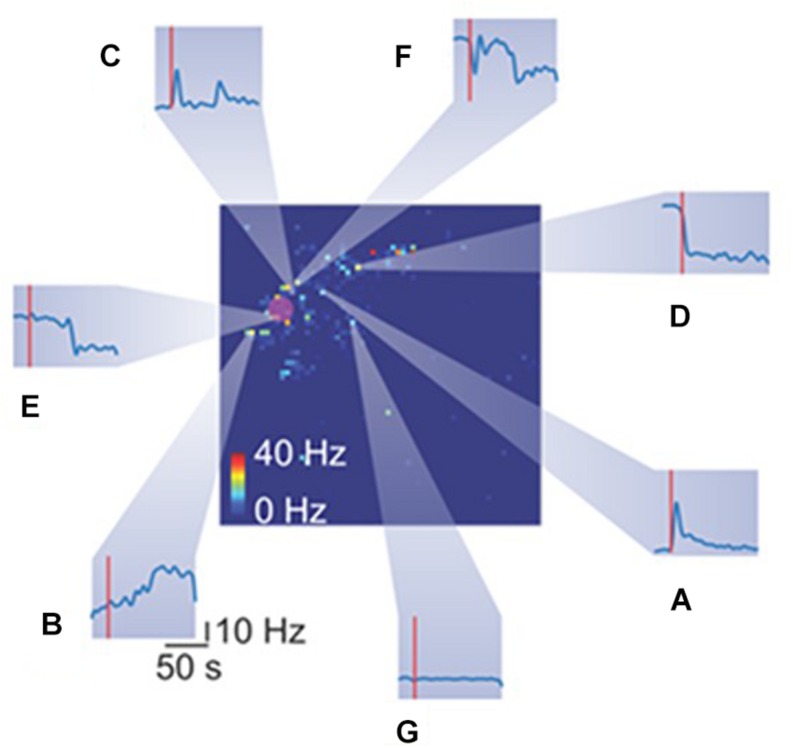
Heterogeneous spiking activity response of retinal ganglion cells (RGCs) to mechanical stimulation. The big square represents the retina spiking activity collected from the HD-MEAs. Each pixel color code the mean firing rate of the RGC recorded from a specific electrode. The red rounded shaded area highlight the indentation area. Each inset (from **A**–**G**) reports the temporal spiking dynamics of different RGCs in response to the same mechanical stimulation (red vertical line represents the stimulation time).

After the characterization of the mechanical properties of the retinal tissue, we investigated the electrophysiological response, signaled through the spiking activity of RGCs, to mechanical stimulation. The overall goal of this section is to validate the potentials of combining sub-micrometer-accurate indentation to HD-MEAs to investigate the effects of mechanical stimulation in neuronal tissue. As stressed above, we simultaneously recorded the spiking activity of hundreds of RGCs with an HD-MEAs system while locally indenting the photoreceptor layer (see Figure 5) to examine the RGCs firing rate spatio-temporal distribution. Next, after identifying the responding RGCs, we checked whether the transduction of mechanical cues was a prerogative of a specific subtype of RGCs, namely ON, OFF, or ON-OFF cells.

### HD-MEAs Recordings

The HD-MEAs can sample the bioelectrical activity of hundreds of RGCs over a large portion of tissue. Upon application of a localized mechanical stroke with the spherical tip of the indentation probe, we focused our attention on possible firing-rate modulations evoked at the pan-retinal scale. Although kept in dark conditions, RGCs are always active, and their spiking activity does not exhibit particular spatio-temporal structures. However, depending on the firing patterns elicited in response to an alternating sequence of black and white flashes that visually stimulate the photoreceptor layer, ON, OFF, and ON-OFF RGCs can be distinguished. RGCs not ascribable to any of the mentioned types were grouped in a fourth class (NC).

We conducted experiments on seven retinae by indenting the photoreceptor layer at four depth (10-20-30-60 μm) and in three retinae we were able to detect mechanically induced responses in the spiking activity of >100 RGCs over >300 RGCs considered for each retina, see Table 1.

**TABLE 1:** Summary of mechanical protocols that determined a variation in the firing rate in retinal ganglion cells.

**Retina ID**	**Amplitude (μm)**	**Depth (μm)**	**Frequency (Hz)**	**Max Load(μN)**	**# of RGCs activated**	**Total RGCs**
R1	/	30	/	5	154	346
	1	30	0.1	1.5	145	346
R2	/	20	/	0.3	102	425
R3	/	60	/	34	129	308

*All the tested mechanical stimulation conditions with an indication of successful/unsuccessful induced responses are included in Figure 8.*

Mechanical stimulation evokes in a subset of RGCs a plethora of different responses, as shown in Figure 5. Heterogeneous responses include, but are not limited to, increase in firing rate at the onset (Figure 5A), at the offset (Figure 5B) or both (Figure 5C) of the stimulation time-interval. Moreover, we observed a drop in the firing rate at onset (Figure 5D) or offset (Figure 5E). Finally, while we recorded a few RGCs with rebounding activities (Figure 5F), the large fraction of RGCs was insensitive to the mechanical stimulation (Figure 5G).

### Mechanical Stimulation Determine Correlated Spiking Activity

**FIGURE 6:**
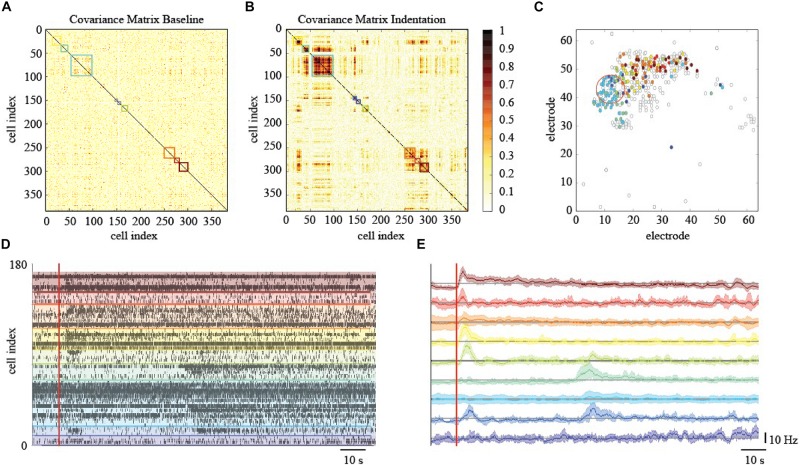
Effects of localized mechanical stimulation onto retinal ganglion cells (RGCs) spontaneous spiking activity. **(A)** Reordered correlation matrix revealing clusters of correlated RGCs during the indentation time interval (color-coded blocks). **(B)** Correlation matrix of the basal activity reordered according to the clustering in **(A)** reveals a different arrangement of correlations. **(C)** Position of RGCs modulated (colored dot) or not (white dot) by the mechanical stimulation. **(D)** Raster plot of the RGCs spiking activity within each cluster (separated by colored lines) during the indentation time interval (red vertical line, onset). **(E)** Z-scored RGCs firing rate during indentation (solid line, mean; shaded area, s.e.m.). Color codes for cluster membership in all panels.

To elucidate and describe the effects of a localized mechanical stimulation at the pan-retinal scale, we clustered together RGCs whose spiking activity was correlated or anti-correlated during the indentation time-window (Figure 6A). Interestingly, this approach reveals the presence of correlated RGCs clusters during the indentation time-window that exhibit the following features. First, their arrangement is not preserved in the basal recording, i.e., the time interval preceding the stimulation onset, where the within-cluster correlation sharply drops (Figure 6B). Second, the clusters of correlated RGCs are well-segregated in space and located in the surrounding of the indentation spot up to a millimeter distance (Figure 6C). Third, as shown in the raster plot of Figure 6D, mechanically evoked responses resemble the one obtained with visual stimulation despite their significantly longer time-scale of activation (tenths of seconds instead of tenths milliseconds, respectively). Fourth, the RGCs spiking activity within the clusters detected is mainly characterized by a prolonged increase in their firing rate lasting a few seconds (see Figure 6E). Nevertheless, inhibition was observed in a very few RGCs, as we will discuss more in details in the next paragraph. Similarly to the ON-OFF classification obtained in response to visual stimuli, we could distinguish between RGCs that activated only to pressure onset (reddish clusters), at pressure release (greenish clusters) or to both (blueish clusters). Fifth, these response delays are consistent with the relative position of RGCs from the indentation spot (in Figure 6C colored dots represents and highlights the RGCs that showed a response modulated by the mechanical indentation vice-versa the white dots). These features were observed in the three retinas who effectively responded to the mechanical stimulation, (see Supplementary Figure S1) whereas the spiking activity of the remaining retinas during the mechanical stimulation time interval remained indistinguishable from their basal. Despite these unsuccessful trials (see later for dedicated discussion), our results still indicate that the retina can convey information about the physical world within the RGCs spike trains and that mechanical stimulation can perturb the spiking activity over extended regions of the retina.

### Processing of Mechanical vs. Visual Sensory Inputs

**FIGURE 7:**
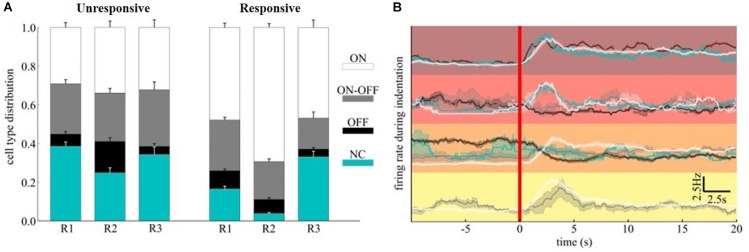
ON-OFF classification of mechanically evoked spiking activities. **(A)** Distribution of ON, ON-OFF, OFF and NC RGCs in clusters associated (“responsive to stimuli”) or not (“unresponsive to stimuli”) to mechanically evoked responses. **(B)** The top four clusters of Figure 6E were further decomposed according to cell type. The red color vertical line marks the starting time of indentation.

Although the time-scales of mechanically evoked responses are not comparable to visual ones, we tested whether the functional subpopulation of RGCs, namely ON-/OFF- or ON-OFF-type, were equally modulated. We functionally classified the RGCs within the relevant clusters detected by quantifying the bias index of the spiking response to white and black full-field visual stimuli (see section “Materials and Methods”). Interestingly, in our experimental conditions of scotopic luminance, the OFF-RGCs response dominates over ON-RGCs response in amplitude while non-classified units (NC) were barely sensitive to visual stimuli with a minor, non-significant, preference to white flashes (NC-type). To assess whether a subpopulation would be primarily involved in conveying mechanical information, we computed the average composition of ON, OFF, ON-OFF, and NC-type RGCs in the clusters associated (“responsive to stimuli”) or not (‘unresponsive to stimuli”) to any mechanically evoked response (Figure 7A). While the cell-type distribution in “unresponsive to stimuli” clusters was fairly similar across the retinae, the “responsive to stimuli” clusters consist of at least 15% more ON-RGCs than the “unresponsive to stimuli” counterparts. Thus, the different cell-type distribution indicates a fundamental contribution of the ON information pathway compared to the others considered in our analysis. Given that our clustering metric is sensitive to both correlated and anti-correlated activities, we investigated whether by further dividing the “responsive to stimuli” clusters according to the ON-OFF classification of RGCs we could reveal additional response features to the mechanical stimuli. This was performed by comparing the firing rate during mechanical (Figure 7B) stimulation.

**FIGURE 8:**
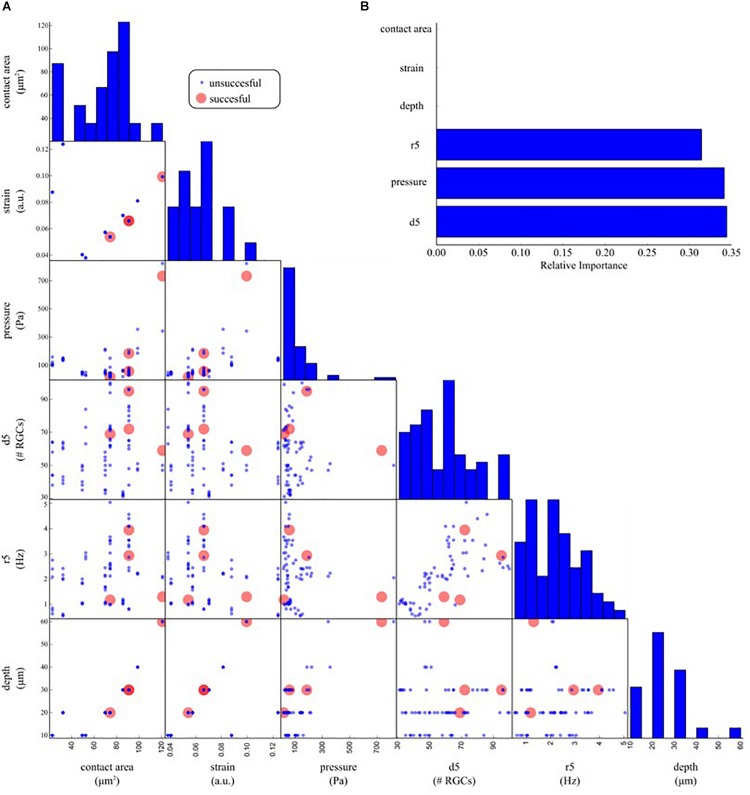
Determinants underlying the physiological response upon indentation. **(A)** Off-diagonal: pair-wise scatter plots of physical (contact area, strain, pressure, depth) and electrophysiological (d5: average RGC density within five electrodes, r5: average RGC firing rate within five electrodes) predictors of an electrophysiological response to mechanical stimulation. Each scatter plot depicts whether a mechanical indentation induced modulation in the RGC firing rate (successful trial, red dots) or not (unsuccessful trial, blue dots) as a function of a pair of predictors. Diagonal: histogram of the distribution of each predictor. **(B)** Relative importance of physical and electrophysiological features in a decision tree model trained to predict successful and unsuccessful trials based on the data in **(A)**. The trained model suggests that local density (d5), pressure, and local firing rate (r5) are the most effective predictors used by the decision tree model to split the successful from the unsuccessful trials.

To investigate what are the most crucial parameters that can give rise to a modulation of the spontaneous activity of RGCs upon mechanical stimulation we organized our data with a machine learning approach. Figure 8A shows a pairwise scatter matrix of the electrophysiological and physical parameters (predictors) involved in the indentation experiments that shows for each condition whether the mechanical stimulation gave rise to a modulation in the firing activity of RGCs comparable to the illustrative example of Figure 6A (successful, red dot) or the mechanical stimulation did not affect the retinal baseline firing (unsuccessful, blue dots). Based on these observations, we trained a decision tree to learn a set of if-then-else rules arranged in a tree-like fashion that splits the parameter space into successful and unsuccessful regions, given the values of predictors. After training, we can interpret what are the rules that the model has learned to identify successful and unsuccessful trials by looking at the structure of the tree. Indeed, by computing how often a predictor is used in the tree to split the data and how much the predictor decreases the impurity (i.e., the fraction of successful and unsuccessful trials before and after the split) we can estimate how important such a feature is. As shown in Figure 8B, according to the decision tree model, the local density of RGCs (d5), the pressure applied (pressure) and the local firing rate (r5) are the ones that most effectively provide information on whether or not a given indentation can induce modulations in the spontaneous firing rate of RGCs.

## Discussion

In this work, we developed an experimental platform combining a depth-controlled force transducer with a planar HD-MEAs device. This platform allows probing contact force effects on the activity of brain circuits determined by large-scale neuronal activity recordings. To demonstrate this technology, we investigated the effects of mechanical stimuli on the electrophysiological response to visual stimuli of explanted mice retinae. We simultaneously recorded the activity of many RGCs, and we investigated the effects on the light responses of three RGC subtypes, namely ON, OFF, and ON-OFF RGCs.

By performing the first depth-controlled frequency-domain indentation tests on mouse retinae ever reported in the literature, we could observe that, in the 0.1–10 Hz range, both apparent storage and loss moduli increase with indentation frequency – a viscoelastic behavior that was previously observed as a stress relaxation and in bulk rheology experiments (Lu et al., 2006; Zhang et al., 2018). A direct comparison with other viscoelastic measurements is unfortunately not possible, because the latter have been either performed on a different kind of samples (human or pig retina) or at nano-scale (single cells, or cell monolayers), which give rise to very different results. Quantitatively, it is interesting to note that, in terms of apparent storage modulus at low frequencies, our findings are in good agreement with Franze et al. (2011) even if the measurements are performed under different indentation protocols. Moreover, Figure 3 shows that both *K*′ and *K*″ increase with increasing frequency of deformation in a manner consistent with numerous previous studies on biological tissues (Mahaffy et al., 2004; Hrapko et al., 2005; Temple et al., 2016; Burton et al., 2017). The trend of K′ and K′ over frequencies depends on the relaxation time spectrum (Deshpande, 2018). Within the frequency range investigated here, the apparent storage modulus K′ always exceeds the loss modulus K′ indicating a predominant elastic response of the material. Moreover, since K′ keeps increasing with frequency, the material relaxation frequency is expected to be outside and to the right of the 0.1–10 Hz range investigated.

However, the high value of *Tan*(Φ) suggests that it is crucial to mechanically characterize the retina by considering both the elastic and viscous contribution. Even if the viscosity is not negligible, the predominance of the elastic behavior of the retina compared to its viscous one is confirmed. From the values reported in Figure 4, it is possible to observe slight variations in local viscoelasticity; however, our findings, obtained by scanning the surface of the sample with 50 μm spatial resolution are not sufficient to evaluate the local heterogeneity of the tissues. Therefore, in the future, to accurately investigate the relationship between the inner retinal morphological structure and its mechanical heterogeneity, one should perform high-resolution viscoelasticity maps.

Through the combination of HD-MEAs system with micro-indentation, we studied the modulation of RGCs spiking activity of explanted mice retinas in response to mechanical micro-stimulations of their photoreceptor layer. Our preliminary findings suggest that it is possible to determine a correlation between the mechanical stimulation of the retina and its electrical signaling. Specifically, we observe an increased firing rate during the indentation time-interval mostly imputable to ON-RGC. In agreement with Rountree et al. (2018), we found that the mechanical stimulation of explanted retinas elicits spatially localized retinal responses similar to light-evoked one, under specific mechanical condition - indentation depth >20 μm and indentation strain ε > 0.05 (Lin et al., 2009). However, Rountree et al. (2018) reported that the mean pressure able to mechanical stimulate the retina is 0.69 kPa, whilst in our depth controlled experiments we found that the minimum pressure to mechanically stimulate the retina is of only 0.02 kPa, possibly meaning that the indentation depth and the sphere radius are the most crucial parameters that can give rise to a light-evoked response. Our experimental data show that the retinal circuit can convey information on mechanical stimuli by modulating RGCs activity, and thus the same retinal circuit’s output of encoded visual stimuli. Recordings of mechanically induced RGCs response show a longer delay from stimuli (tens of seconds) compared to delays of light-evoked visual responses (milliseconds). This response latency could be due to the slow indentation profiles used in our measurements or to the fact that mechanical stroke is focused on the photoreceptor layer and the stress propagates in a certain time scale until the ganglion cell layers. In this context, in the future, it would be interesting to tweak the indentation profile (e.g., indentation speed and oscillation amplitude and frequency) in order to modulate and to accurately study the propagation of the mechanically evoked response in the tissue.

Given that our results indicate that mechanical stimuli are likely encoded in RGCs activity, another intriguing possibility, instead, is that RGCs convey to downstream areas multiple sensory information (Križaj, 2016), and in particular intraocular pressure (Kalapesi et al., 2005). In this scenario, different messages might be conveyed through the same pathway by using distinct time-scales as information carriers.

Interestingly, we also observed that the mechanically evoked responses are spatially distributed in the surrounding of the indentation location and not only under the stimulated point, possibly indicating a slow horizontal propagation of the mechanically evoked bioelectrical perturbation across the retinal tissue. This result can be consistent with the propagation of calcium waves in Muller’s cells triggered by pressure signals (Newman and Zahs, 1997). Such calcium waves, indeed, can impact on retinal ganglion cell spiking activity by modulating their response to visual stimulation (Newman and Zahs, 1998). In addition, the modulation of basal firing rate we observed in this work is consistent with the time-scale of Muller’s cells calcium waves (Newman and Zahs, 1997; Lindqvist et al., 2010).

However, the biological origin of the modulation in firing rate observed in this work is not limited to activation of Muller’s cells but may be attributed to other mechanisms acting together, as direct modulation of photoreceptors and horizontal cells (Tan et al., 2006).

Ultimately, our findings could possibly cast a shadow on the effects of using weights (e.g., metal anchors) usually employed in electrophysiological experiments on explanted retinas. Indeed, in the light of what demonstrated in this work, we cannot exclude the presence of distortion in the retinal signal induced by the constant pressure produced by external weight commonly used during recording sessions for keeping the tissue attached to the multi-electrode array surface. Some possible side-effects have been already reported in literature (Fujii et al., 2016) and need to be further investigated in order to disentangle whether these differences are related to an electrode-tissue coupling issue (i.e., collected signals vary depending on the anchor weight because the distance between the electrodes and the RGCs is different) or if the constant force applied on top of the retina interferes with the visual processing pathway consequently modifying the RGCs output.

Moreover, it is worth to mention that several factors could explain the unsuccessful induced responses. First of all, our intent was mainly to explore whether the retina could respond to mild mechanical stimuli to avoid second order effects mainly due to retinal damage. As a consequence, we decided to deliver shallow mechanical stimuli, and we expected to observe more unsuccessful than successful trials. A second major source of inter-trial variability is the location of the indentation. Specifically, we targeted areas of the retina in which the coupling with the MEA was optimal. However, such a heuristic criterion may lead to the stimulation of retina patches that are, *per se*, functionally, and structurally different locally. Moreover, we were not able to precisely identify in which region of the sample we were indenting (center vs. periphery, medial vs. lateral, frontal vs. nasal). Of course, the electrophysiological response of the retina can differ between those regions, and it will be of utmost interest, to investigate in the future if, for example, the unsuccessful cases are related to the different regions of the retina. Third, one of the main challenges of performing these experiments on live tissue is related to the necessity to keep the tissue stable and alive over the course of the measurements. The entire stimulation protocol takes around 20 min per location. Therefore those time scales do not allow us to map the entire retina *in vivo* and long-timescale could compromise the vitality of the tissue inducing and inter and intraretinal variability. Another factor of variability between the retinae could be related to the preparation of the biological sample itself and the immobilization of the sample on the multi-electrode array surface. The adhesion of the retina onto the multi-electrode array surface is extremely important in order to avoid to stimulate the floating tissue rather than probing the tissue stiffness.

## Conclusion

In conclusion, we have introduced a new technique that allows the analysis of the electrical signals cascade occurring through neuronal networks under controlled mechanical stimulation and that is able to reliably measure the viscoelastic properties of soft biological samples. In the future, the combination of HD-MEA system with micro-indentation could offer a unique opportunity to investigate the effects of mechanical stimulation on the electrophysiological activity of neuronal tissue also at single-cell resolution and over a wide portion of biological tissue such as stem cells (Vogel and Sheetz, 2006; Reilly and Engler, 2010; Yim and Sheetz, 2012; Vining and Mooney, 2017), heart (Terracio et al., 1988; Franz et al., 1989; Kohl et al., 2006), lungs (Wirtz and Dobbs, 2000), muscle (Hunt, 1952; Crago et al., 1976) and skin (Chouvardas et al., 2008). Finally, since mechanical stress can modulate physiological processes at the molecular and cellular level, we expect that this tool will support a significant step forward in gaining new insights on the relationship between altered mechanosensitive signaling, stiffness, and pathologies.

## Data Availability Statement

All raw and processed data of this study are available on reasonable request from the corresponding author.

## Ethics Statement

The animal study was reviewed and approved by the Institutional IIT Ethics Committee and by the Italian Ministry of Health and Animal Care (Authorization number 110/2014-PR, December 19, 2014).

## Author Contributions

LB and DI designed the research. HH and AM performed the preliminary experiments. MM, DL, and FB performed the experiments and analyzed the data. SZ provided the python algorithm for the trigger on the stimulation time-window. MM, DL, FB, DI, and LB wrote the manuscript. All authors critically revised the manuscript for intellectual content and approved the final manuscript.

## Conflict of Interest

DI declares a potential conflict of interest as founder, shareholder, and advisor of Optics11. The remaining authors declare that the research was conducted in the absence of any commercial or financial relationships that could be construed as a potential conflict of interest.
