# Supplementary material for: Investigating the Effects of Mechanical Stimulation on Retinal Ganglion Cell Spontaneous Spiking Activity
Source: Front Neurosci. 2019 Sep 27;13:1023. doi: 10.3389/fnins.2019.01023 (PMC6776634; doi:10.3389/fnins.2019.01023)
Supplement: Supplementary file 1 [file Data_Sheet_1.docx]

**Supplementary figure**

**
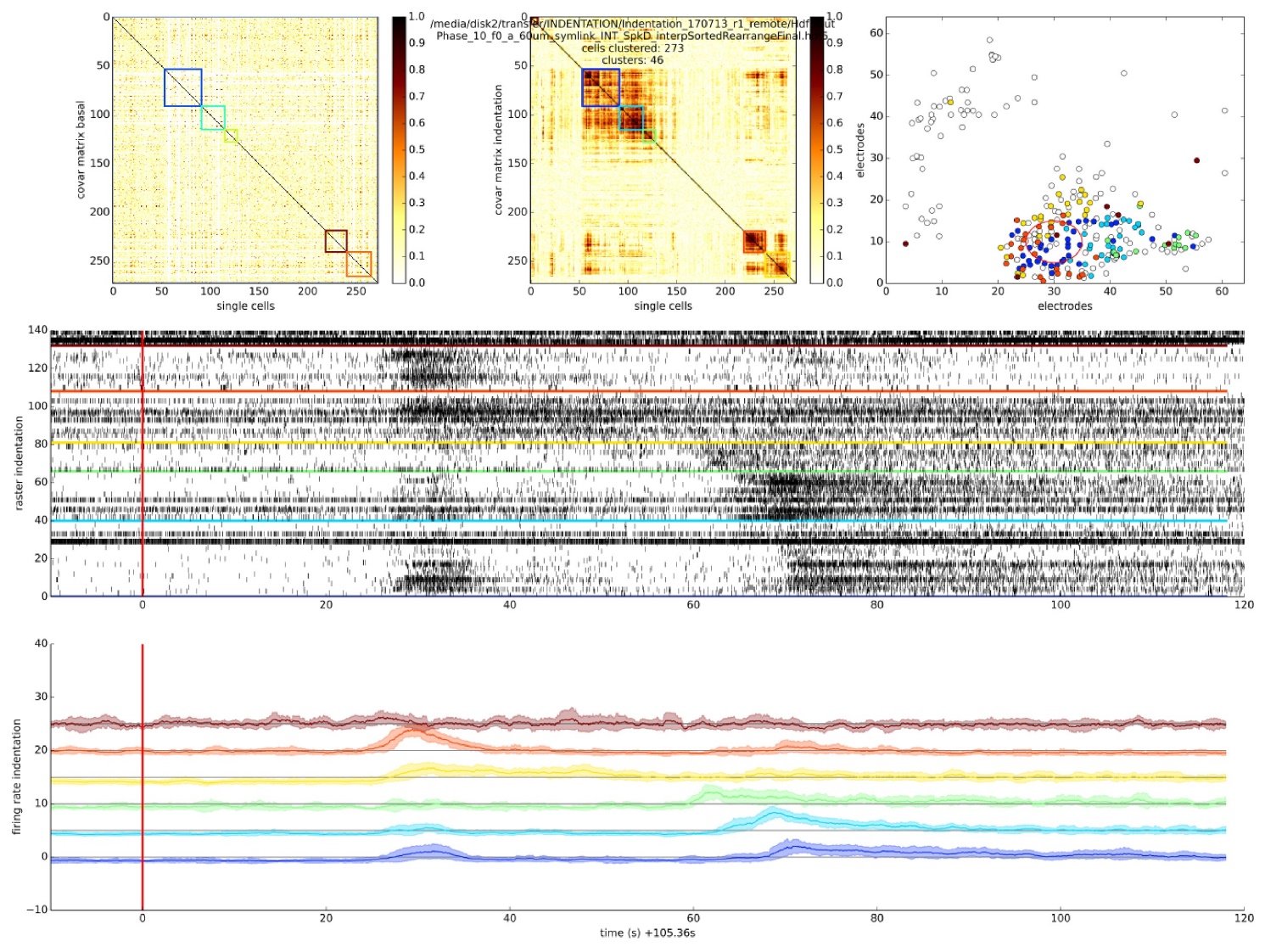
**

**Figure S1: Additional example (c.f.r. Figure 6) of mechanical stimulation effects onto retinal ganglion cells (RGCs) spontaneous spiking activity. (A) Reordered correlation matrix revealing clusters of correlated RGCs during the indentation time interval(color-coded blocks). (B) Correlation matrix of the basal activity reordered according to the clustering in (A) reveals a different arrangement of correlations. (C) Position of RGCs modulated (colored dot) or not (white dot) by the mechanical stimulation. (D) Raster plot of the RGCs spiking activity within each cluster (separated by colored lines) during the indentation time interval (red vertical line, onset). (E) Z-scored RGCs firing rate during indentation (solid line, mean; shaded area, s.e.m.). Color codes for cluster membership in all panels.**
